# Stand composition shapes canopy structure, competition, and soil properties in virgin oriental beech forests

**DOI:** 10.1038/s41598-026-48111-3

**Published:** 2026-04-14

**Authors:** Mojtaba Azaryan, Kambiz Abrari Vajari, Azade Deljouei, Marina Viorela Marcu, Seyed Mohammad Moein Sadeghi

**Affiliations:** 1 Department of Forestry, Faculty of Natural Resources, Lorestan University, Khorramabad, Iran; 2 School of Forestry, Northern Arizona University, Flagstaff, AZ USA; 3 Department of Forest Engineering, Forest Management Planning and Terrestrial Measurements, Faculty of Silviculture and Forest Engineering, Transilvania University of Brasov, Şirul Beethoven 1, Brasov, 500123 Romania

**Keywords:** *Fagus orientalis*, Forest–soil coupling, Reference forests, Structural heterogeneity, Temperate forests, Tree competition, Ecology, Ecology, Environmental sciences

## Abstract

Forest stand composition influences ecosystem structure and functioning, yet its integrated effects on canopy architecture, competition intensity, and soil processes are rarely evaluated under undisturbed conditions that provide essential silvicultural reference states. The virgin oriental beech forests of the Hyrcanian region in northern Iran represent one of the last remaining temperate broadleaf forest systems worldwide that have developed without logging, grazing, or silvicultural intervention, providing a rare natural reference for examining coupled above- and belowground processes. We compared pure and mixed beech stands by quantifying canopy structural attributes, competition indices, and key organic and mineral soil properties. Pure stands exhibited larger trees, stronger vertical canopy organization, and significantly higher competition intensity, whereas mixed stands supported greater microbial biomass and respiration in the mineral soil despite lower competitive pressure. Correlation and redundancy analyses revealed strong linkages between canopy structure, competition intensity, and soil properties, with clear multivariate separation between pure and mixed stands. These findings demonstrate that stand composition reorganizes the coupling between canopy structure, competition intensity, and soil processes, highlighting the importance of integrating above- and belowground components to understand ecosystem functioning in undisturbed forests. By leveraging one of the few remaining virgin temperate beech forest landscapes, this study provides a basis for targeted silvicultural and restoration strategies by identifying when mixed-species stands enhance soil microbial functioning and when pure beech stands promote larger tree development and greater vertical canopy stratification.

**Supplementary Information:**

The online version contains supplementary material available at 10.1038/s41598-026-48111-3.

## Introduction

Forest stand composition and structural organization are central determinants of ecosystem functioning, regulating resource acquisition, competitive interactions, and soil processes across forested landscapes^1,2^. In temperate broadleaf forests, variation in canopy architecture and tree size distributions influences light interception^3^, litter inputs^4^, microclimatic conditions^5^, and belowground nutrient cycling^6^, thereby shaping forest productivity and biogeochemical dynamics^7^. Understanding how differences in stand composition and structural organization propagate through these interconnected above- and belowground pathways is therefore essential for predicting forest productivity, soil functioning, and biogeochemical feedbacks to management and environmental change.

Stand composition represents a key dimension of forest structural organization, with pure and mixed stands often differing markedly in canopy architecture^8^, tree size distributions^9^, and spatial patterns of resource use^10^. Mixed stands commonly exhibit greater structural heterogeneity due to differences in species-specific growth strategies and crown traits, whereas pure stands are typically characterized by more uniform canopy layers and size hierarchies^11^. These structural contrasts influence the intensity and spatial distribution of competitive interactions, affecting how resources are captured and allocated among trees^12^. Tree size inequality, crown development, and spatial arrangement collectively determine competitive intensity for light, water, and nutrients, with cascading effects on growth patterns and carbon allocation^13^. In structurally simple stands, competition may be more intense and vertically integrated, whereas structurally heterogeneous stands can exhibit more diffuse competitive interactions and greater functional complementarity^14^. Quantifying competition using structurally informed indices therefore provides an effective framework for linking stand structure to ecosystem functioning^15^.

Structural and competitive constraints among stands have important consequences for soil processes, as canopy structure governs the quantity, quality, and spatial distribution of organic inputs and microenvironmental conditions at the forest floor^16,17^. Differences in crown architecture^18^ and litter production^19^ can alter organic layer development and influence microbial activity, nutrient availability, and carbon stabilization in the mineral soil. While the organic layer reflects relatively short-term responses to canopy processes, mineral soil properties integrate longer-term interactions among vegetation, microorganisms, and soil physical characteristics^20^, making both compartments essential for understanding structure–soil linkages. Despite growing recognition of these structure–soil linkages, canopy structure, competition, and soil properties are frequently examined in isolation, limiting insight into their combined effects across contrasting stand types. Addressing this limitation requires reference forest systems in which variation in stand composition can be evaluated under natural conditions, with minimal confounding environmental influences.

Virgin oriental beech (*Fagus orientalis* Lipsky) forests of northern Iran constitute a globally rare reference system for examining coupled above- and belowground processes in temperate broadleaf forests^21^. These forests represent some of the last remaining remnants of extensive virgin temperate forest in the Western Palearctic^22,23^, where long-term stand development has proceeded under minimal direct human disturbance. In contrast to most temperate forests in Europe and North America—where centuries of management have largely eliminated true old-growth conditions—pure and mixed beech stands in the Hyrcanian region persist in close spatial proximity. This unique landscape configuration provides an exceptional opportunity to evaluate the effects of stand composition while minimizing confounding influences of climate, topography, and soil parent material, especially in a small patchy virgin area. Such conditions are increasingly uncommon at the global scale, yet they are essential for isolating structural and competitive controls on soil processes under natural conditions. Despite the high ecological and conservation value of these forests, integrated studies that simultaneously link canopy structural attributes, competition intensity, and both organic and mineral soil properties across pure and mixed virgin stands remain scarce.

While previous studies in Hyrcanian beech forests have examined stand structure or soil processes independently, and have demonstrated the influence of species composition on nutrient cycling^24,25^ and structural dynamics^26^, these components have rarely been evaluated within a unified framework. In particular, the extent to which canopy structural organization and competition intensity jointly regulate soil physicochemical and biological properties under undisturbed conditions remains poorly resolved. Addressing this gap requires integrative approaches that explicitly link aboveground structural complexity and competitive interactions with belowground ecosystem functioning.

In this study, we examine coupled variation in canopy structure, competition intensity, and soil properties across pure and mixed virgin oriental beech stands. Specifically, we aim to (1) quantify differences in canopy structural attributes and competition intensity between stand types, (2) evaluate corresponding responses in organic and mineral soil physicochemical and biological properties, and (3) assess multivariate relationships between canopy structure, competition, and soil properties. Based on current ecological understanding, we formulated the following hypotheses: (H1) mixed beech stands exhibit greater structural heterogeneity, whereas pure stands are characterized by larger tree dimensions and higher competition intensity due to stronger conspecific interactions; (H2) soil physicochemical and biological properties differ between stand types, with mixed stands supporting higher microbial activity and nutrient availability as a result of differences in litter quality and species composition; and (H3) canopy structural attributes and competition intensity jointly explain variation in soil properties, indicating a coupling between above- and belowground ecosystem functioning. By integrating above- and belowground components under true reference conditions, this study provides a mechanistic and multivariate assessment of forest structure–soil coupling, offering rare benchmarks for how stand composition reorganizes ecosystem functioning in undisturbed temperate forests.

## Methods

### Site description and stand selection

The study was conducted in virgin *Fagus orientalis* Lipsky stands located in the western part of the Hyrcanian temperate forest region, within the Asalem Forest (Guilan Province), northern Iran (37°39′12″–37°39′26″ N; 48°45′07″–48°45′35″ E). The regional climate is humid temperate, characterized by relatively high annual precipitation and moderate temperatures. Mean annual precipitation is approximately 1,300 mm and is distributed throughout the year, with no pronounced dry season. Mean annual air temperature is about 8.5 °C, with minimum and maximum annual averages of 4.5 °C and 16.0 °C, respectively, resulting in cool winters and mild summers^27^.

Study sites were selected from national inventories of virgin forests conducted by the Iranian Research Institute of Forests and Rangelands (IRIFR) in the 1990s^28^. These inventories identified forest remnants lacking evidence of historical logging, road construction, grazing, or silvicultural intervention and meeting national criteria for classification as virgin forest. Two stand types were selected for analysis: pure beech stands and mixed beech stands. The selected pure and mixed stands occur in close spatial proximity and were chosen to ensure comparable environmental conditions, including elevation, slope, aspect, climate, and soil parent material. All selected stands have remained under long-term protection and exhibit high structural integrity, including the presence of large trees, vertically developed canopies, and undisturbed soil profiles.

### Field sampling

According to the national inventory of virgin forests conducted by the IRIFR, approximately six hectares of virgin *F. orientalis* forest with both pure and mixed stand compositions are officially recognized in Guilan Province^28^. From this area, four hectares—comprising 2 ha of pure beech stands and 2 ha of mixed beech stands—were classified as being in the optimal stage of stand development and were selected for field investigation. Within each stand type, fifteen square sample plots (20 m × 20 m; 400 m^2^) were established using a random placement design. Plots were distributed with a minimum separation distance of 60 m between plot centers to minimize spatial autocorrelation and to reduce overlap among canopy and rooting zones. This design resulted in a total of 30 plots, equally allocated between pure and mixed beech stands, ensuring comparable sampling intensity across stand types. All plots were located within a single continuous forest patch and were deliberately positioned away from stand edges, recent natural canopy openings, and atypical microsites to ensure that sampled conditions were representative of mature, closed-canopy forest structure and to minimize variability associated with landscape fragmentation or patch-level heterogeneity.

To verify the assumption of independence among plots, spatial autocorrelation was evaluated using Moran’s I^29^ based on plot centroid coordinates. Significance was assessed using permutation tests. Results indicated no significant spatial autocorrelation among plots (*p* > 0.05), supporting the validity of treating plots as independent observational units.

### Canopy structural parameters and competition index

Canopy structural attributes were quantified at the plot level to characterize tree size distribution, vertical canopy organization, and the competitive environment. All live trees with a diameter at breast height (DBH) ≥ 7.5 cm within each plot were included in the analysis. For each tree, DBH (cm) and total tree height (m) were measured. Trunk height was defined as the vertical distance from the ground surface to the base of the lowest live primary branch. Crown length was calculated as the difference between total tree height and trunk height. Crown projection area (CPA, m^2^) was estimated from crown radius measurements, assuming an elliptical crown geometry. The slenderness coefficient (SC) was calculated as the ratio of tree height to DBH and used as an indicator of tree form and mechanical stability under competitive conditions^30^.

To quantify competitive intensity within each plot, a competition index based on species-specific wood density (hereafter CI_wood_)^31 was used^. This index integrates tree size and functional traits to capture differences in competitive capacity among co-occurring species. The competition index was computed as:

1 $$\:CI_{{wood}} = \sum\limits_{{i = 1}}^{n} {\left( {DBH_{i}^{2} \times \:h_{i} \times \:\sigma \:_{i} } \right)}$$

where ∑ denotes the summation operator over all trees *i* within a plot (*i* = 1 to *n*), $\:{DBH}_{i}$is the diameter at breast height of tree *i*, $\:{h}_{i}$is the total height of tree *i*, and $\:{\sigma\:}_{i}$represents the species-specific wood density assigned to tree *i*. Wood density values were obtained from published global databases^32^. This formulation assumes that tree sizes (DBH × h) provides a proxy for competitive influence, while wood density reflects interspecific differences in growth strategy, mechanical strength, and resource-use efficiency. Unlike conventional competition indices that rely primarily on tree size or spatial relationships^33–35^, incorporating wood density introduces a functional trait component linked to species-specific growth strategies and resource-use efficiency^36–38^. Wood density is closely associated with carbon allocation patterns^39,40^, mechanical support, and life-history strategies, and thus provides a biologically meaningful proxy for differences in competitive ability among species^41^. Integrating wood density with tree size therefore allows CI_wood_ to capture both structural dominance and interspecific functional variation, which is particularly relevant in mixed-species stands where trait differences influence competitive interactions and ecosystem processes^37^. Plot-level CI_wood_ values were obtained by summing individual tree contributions, providing an integrated measure of competitive intensity.

To complement this trait-informed metric, we also calculated a spatially explicit competition index using the Hegyi index (hereafter CI_Hegyi_)^33^, which has been widely applied in forest ecology studies to represent local competitive interactions among trees^42–46^. For each focal tree, the CI_Hegyi_ was computed from the DBH of the focal tree, the DBH of its four nearest neighboring trees, and the corresponding inter-tree distances:

2 $$\:CI_{{Hegyi,i}} = \sum\limits_{{j = 1}}^{k} {\frac{{DBH_{j} }}{{DBH_{i} \times \:D_{{ij}} }}}$$

where CI_Hegyi_​ is the competition index of focal tree *i*, DBH_i_ and DBH_j_ are the diameters at breast height of the focal and neighboring trees, respectively, D_ij_ is the distance between trees *i* and *j*, and *k* is the number of nearest neighbors considered (here, *k* = 4). Nearest-neighbor data were recorded irrespective of plot boundaries; thus, neighboring trees located outside the 20 × 20 m plot were included when they belonged to the four closest competitors of the focal tree. This approach reduced edge-related bias and allowed a more realistic representation of local competition. Individual-tree values were subsequently averaged at the plot level to derive a mean CI_Hegyi_ for each plot. The combined use of CI_wood_ and CI_Hegyi_ allows comparison between a trait-informed and a conventional distance-based index, providing complementary perspectives on competitive interactions.

To characterize size inequality within stands, the coefficient of variation (CV) of DBH was calculated at the plot level.

### Soil sampling and laboratory analysis

Soil sampling was conducted separately for the organic layer and the underlying mineral soil (0–20 cm depth). Within each plot, five subsamples were collected—one from the plot center and four from the plot corners—and composited by layer to obtain one representative organic-layer sample and one mineral-soil sample per plot.

The organic layer (forest floor) was sampled by collecting undecomposed and partially decomposed organic material above the mineral soil surface following removal of recent surface litter. Samples were air-dried, gently homogenized, and sieved to 2 mm prior to analysis. Organic-layer pH was determined in a 1:1 (w/v) material-to-water suspension after equilibration, and electrical conductivity (EC) was measured in a saturated extract using a conductivity meter. Total organic carbon (C) was quantified using the Walkley–Black wet oxidation method^47^, total nitrogen (N) was determined by Kjeldahl digestion^48^, and total phosphorus (P) was measured following standard extraction procedures.

Mineral soil samples were collected from the upper mineral horizon after careful removal of the organic layer. Samples were transported to the laboratory on the day of collection, air-dried under ambient conditions, gently crushed, and passed through a 2-mm sieve. Soil texture (sand, silt, and clay fractions) was determined using the hydrometer method^49^. Mineral soil pH and EC were measured in saturated paste extracts. Total C and N were quantified using standard combustion or wet oxidation techniques, and total P was determined using established colorimetric methods. The mineral soil C/N ratio was calculated accordingly. Biological properties of the mineral soil were assessed using fresh field-moist subsamples prior to drying or sieving to preserve microbial activity. Microbial biomass carbon (MBC)^50^, nitrogen (MBN)^51^, and phosphorus (MBP)^52^ were determined using the chloroform fumigation–extraction (CFE) method, a widely applied approach for estimating soil microbial biomass in forest ecosystems (e.g., ^53–56^). Soil samples were fumigated with ethanol-free chloroform for 24 h in a sealed desiccator, followed by extraction with 0.5 M K_2_SO_4_. Non-fumigated samples were extracted in parallel, and microbial biomass was calculated as the difference between fumigated and non-fumigated extracts using standard conversion factors. Microbial respiration was measured as basal soil respiration under controlled laboratory conditions using alkali absorption techniques. Fresh soil samples were incubated at 25 °C for 24 h, and evolved CO_2_ was trapped in NaOH solution and subsequently quantified by titration^57,58^.

### Statistical analysis

All statistical analyses were performed in R (R Core Team, Vienna, Austria), and all figures were generated using *ggplot2*. Plot-level measurements were treated as independent observational units. Prior to analysis, all variables were screened for missing values, outliers, and distributional properties to ensure suitability for parametric analyses. Differences in studied variables and indicators between pure and mixed oriental beech stands were evaluated using unpaired two-sample *t*-tests. Statistical significance was assessed at *p* < 0.05.

Bivariate relationships among canopy structural attributes, competition indices (both CI_wood_ and CI_Hegyi_), and soil properties (organic and mineral layers) were examined using Pearson correlation analysis. Correlation matrices were developed separately for pure and mixed stands to account for stand-specific structure–soil relationships. Results were visualized using correlation heatmaps, in which correlation coefficients were represented by color gradients and magnitude, and levels of statistical significance were indicated using asterisk notation (*p* < 0.05, ** *p* < 0.01, *** *p* < 0.001).

Multivariate relationships between canopy structure, competition intensity, and soil properties were examined using redundancy analysis (RDA) implemented in the *vegan* package. Prior to analysis, candidate variables were screened for multicollinearity and redundancy to prevent inflation of explained variance and to improve ordination interpretability. Multicollinearity was assessed using variance inflation factors (VIFs), and variables with high VIF values were iteratively removed. In addition, strongly correlated variables were identified using pairwise Pearson correlations, and only one representative variable from each highly correlated set was retained based on ecological relevance. This screening resulted in a parsimonious set of predictors representing independent structural and edaphic gradients. All retained variables were standardized (z-score transformation) prior to analysis. Ordination results were visualized using site scores and biplot vectors, with only variables showing the strongest contributions displayed to enhance clarity. Stand types were overlaid using group centroids and 95% confidence ellipses, and the proportion of constrained variance explained by each RDA axis was calculated from canonical eigenvalues. The statistical significance of the RDA model and canonical axes was assessed using permutation tests (*n* = 999 permutations).

## Results

### Canopy structural variables and competition index

**Fig. 1:**
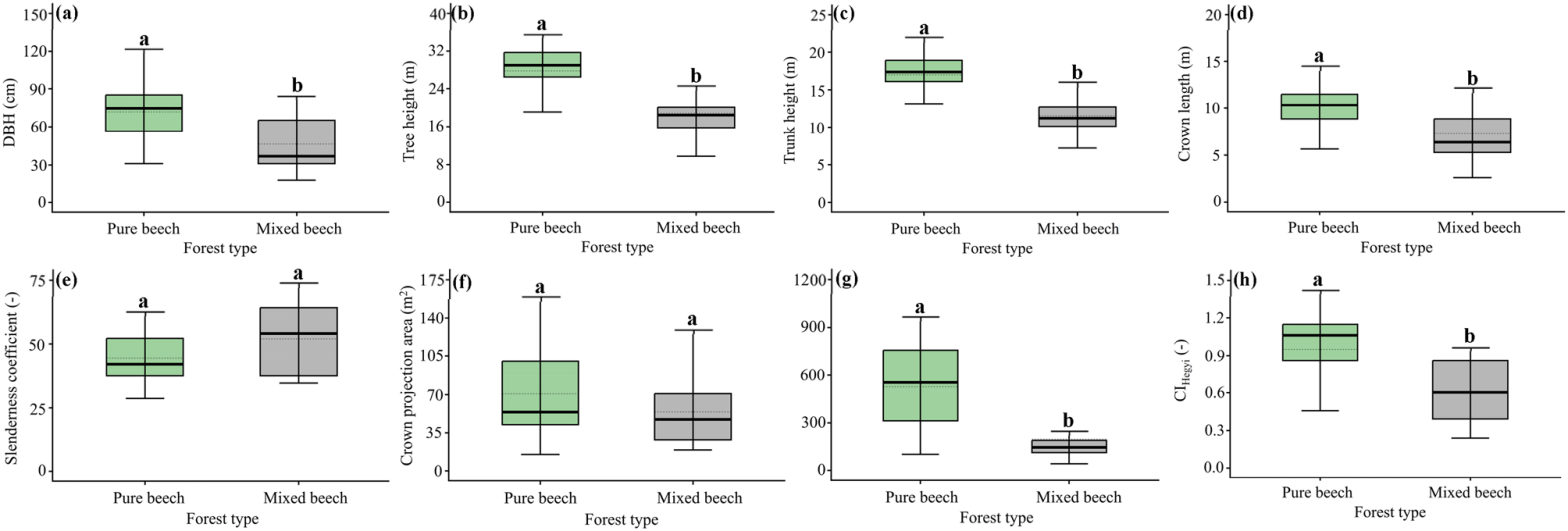
Comparison of canopy structural attributes and competition intensity between pure and mixed oriental beech forests. Boxplots show differences in (**a**) diameter at breast height (DBH), (**b**) tree height, (**c**) trunk height, (**d**) crown length, (**e**) slenderness coefficient, (**f**) crown projection area, (**g**) wood-density–based competition index (CI_wood_), and (**h**) Hegyi competition index (CI_Hegyi_). Boxes represent the interquartile range with the median shown as a solid line; whiskers indicate the minimum and maximum values. Different lowercase letters above boxplots indicate statistically significant differences between forest types based on unpaired t-tests (*p* < 0.05).

Pure and mixed beech stands exhibited significant differences in several canopy structural attributes (Fig. 1; Table 1). Table 1 further shows clear differences in species composition and dominance, with pure stands overwhelmingly dominated by *Fagus orientalis*, whereas mixed stands exhibited a more diverse species composition with lower dominance by any single species. Trees in pure beech stands had significantly larger DBH and greater total height than those in mixed stands (Fig. 1a, b). Trunk height (Fig. 1c) and crown length (Fig. 1d) were also significantly higher in pure stands. In contrast, the slenderness coefficient did not differ significantly between forest types (Fig. 1e). Crown projection area showed no statistically significant difference between pure and mixed stands, although variability was higher in pure beech plots (Fig. 1f). The wood-density–based competition index (CI_wood_) differed markedly between forest types (Fig. 1g). The spatially explicit Hegyi competition index (CI_Hegyi_) showed patterns consistent with the stand-level competition index, with significantly higher competition intensity in pure beech stands compared to mixed stands (Fig. 1h).

**Table 1:** Stand structural characteristics of pure and mixed oriental beech forests.

Stand type	Species	Relative abundance (%)^*^	Relative basal area (%)^*^	Stem density (sph)
Pure beech	*Fagus orientalis*	92.39	98.67	161.7
	*Carpinus betulus*	2.86	0.19	5.0
	*Alnus subcordata*	1.90	1.07	3.3
	*Quercus castaneifolia*	0	0	0
	*Acer velutinum*	0.95	0.03	1.7
	*Acer cappadocicum*	0	0	0
	*Tilia begoniifolia*	0.95	0.02	1.7
	*Prunus avium*	0	0	0
	*Diospyros lotus*	0.95	0.02	1.7
	Total	**100**	**100**	**175.1**
Mixed beech	*Fagus orientalis*	60.58	65.85	138.3
	*Carpinus betulus*	14.60	9.76	33.3
	*Alnus subcordata*	6.57	7.63	15.0
	*Quercus castaneifolia*	2.92	8.61	6.7
	*Acer velutinum*	5.84	4.15	13.3
	*Acer cappadocicum*	1.46	0.61	3.3
	*Tilia begoniifolia*	2.19	1.38	5.0
	*Prunus avium*	2.92	1.92	6.7
	*Diospyros lotus*	2.92	0.09	6.7
	Total	**100**	**100**	**228.3**

^*^ Relative abundance (%) is based on stem density (number of trees per species relative to total stem count), and relative basal area (%) represents the proportion of total stand basal area contributed by each species.

Diameter class distributions further indicated clear differences in size structure between stand types (Fig. S1). Mixed stands exhibited a higher frequency of trees in small- to medium-diameter classes, with a relatively continuous distribution across DBH classes. In contrast, pure beech stands showed a lower frequency of small-diameter trees and a greater representation of large-diameter individuals (Fig. S1).

The coefficient of variation (CV) of DBH was high in both stand types (pure: 79.7%; mixed: 80.7%), indicating substantial size inequality and structural heterogeneity.

### Soil organic layer: chemical properties

**Fig. 2:**
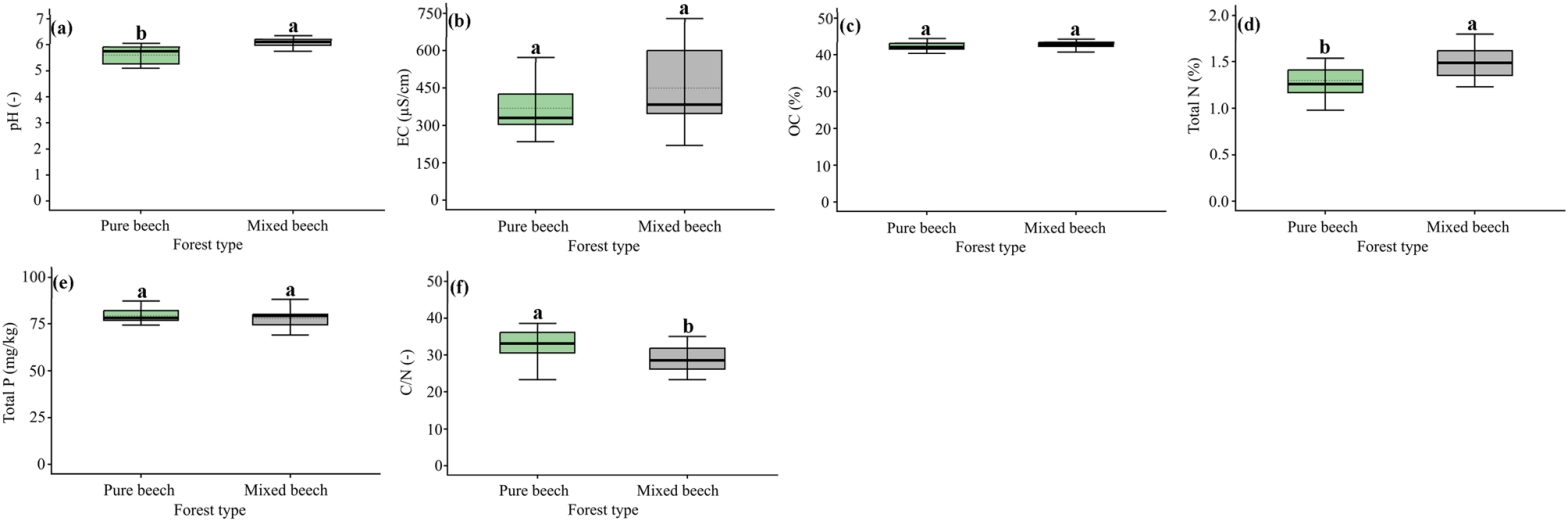
Comparison of organic layer chemical properties between pure and mixed virgin oriental beech forests. Boxplots show differences in (**a**) pH, (**b**) electrical conductivity (EC), (**c**) organic carbon content (OC), (**d**) total nitrogen content, (**e**) total phosphorus, and (**f**) C:N ratio. Boxes represent the interquartile range with the median shown as a solid line; whiskers indicate the minimum and maximum values. Different lowercase letters above boxplots indicate statistically significant differences between forest types based on unpaired t-tests (*p* < 0.05).

Organic layer chemical properties differed between pure and mixed virgin oriental beech stands (Fig. 2). Mixed stands exhibited significantly higher pH and total nitrogen content than pure stands, whereas organic carbon content did not differ significantly between forest types (Fig. 2a, c, d). Electrical conductivity (EC) showed no significant difference between pure and mixed stands, despite higher variability in mixed beech plots (Fig. 2b). Total phosphorus concentrations were similar between forest types and did not differ significantly (Fig. 2e). In contrast, the C:N ratio was significantly higher in pure beech stands (Fig. 2f).

### Soil mineral layer: physicochemical and biological properties

**Fig. 3:**
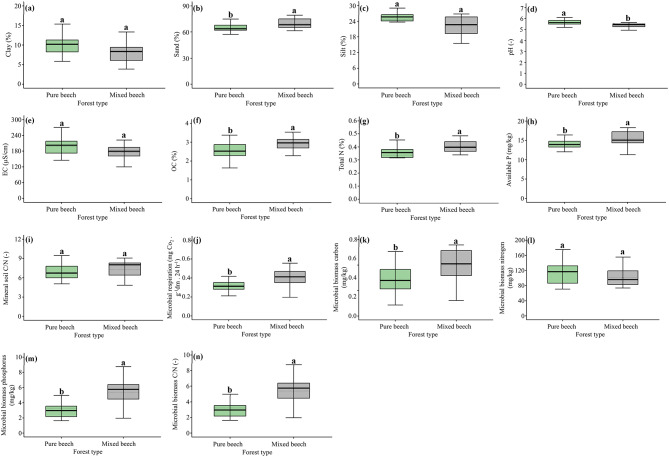
Comparison of mineral soil physicochemical and biological properties between pure and mixed virgin oriental beech forests. Boxplots show differences in (**a**) clay content, (**b**) sand content, (**c**) silt content, (**d**) pH, (**e**) electrical conductivity (EC), (**f**) organic carbon content (OC), (**g**) total nitrogen content (Total N), (**h**) available phosphorus, (**i**) mineral soil C:N ratio, (**j**) microbial respiration (24 h), (**k**) microbial biomass carbon, (**l**) microbial biomass nitrogen, (**m**) microbial biomass phosphorus, and (**n**) microbial biomass C:N ratio. Boxes represent the interquartile range with the median shown as a solid line; whiskers indicate the minimum and maximum values. Different lowercase letters above boxplots indicate statistically significant differences between forest types based on unpaired t-tests (*p* < 0.05).

Mineral soil physicochemical properties exhibited selective differences between pure and mixed virgin oriental beech stands (Fig. 3). Sand content was significantly higher in mixed stands, whereas clay and silt contents did not differ significantly between forest types (Fig. 3a–c). Mineral soil pH was significantly higher in pure beech stands (Fig. 3d), while EC showed no significant difference between forest types (Fig. 3e). Organic carbon and total nitrogen contents were significantly higher in mixed stands compared to pure stands (Fig. 3f, g), and available phosphorus was also significantly greater in mixed beech forests (Fig. 3h). Mineral soil C:N ratio did not differ significantly between forest types (Fig. 3i). In contrast, mineral soil biological properties showed pronounced differences between forest types (Fig. 3j–n). Microbial respiration was significantly higher in mixed beech stands relative to pure stands (Fig. 3j). Mixed stands also exhibited significantly greater microbial biomass carbon and phosphorus, whereas microbial biomass nitrogen did not differ significantly between forest types (Fig. 3k–m). Microbial biomass C:N ratio was significantly higher in mixed beech stands (Fig. 3n).

### Relationships among canopy structure, competition intensity, and soil properties

**Fig. 4:**
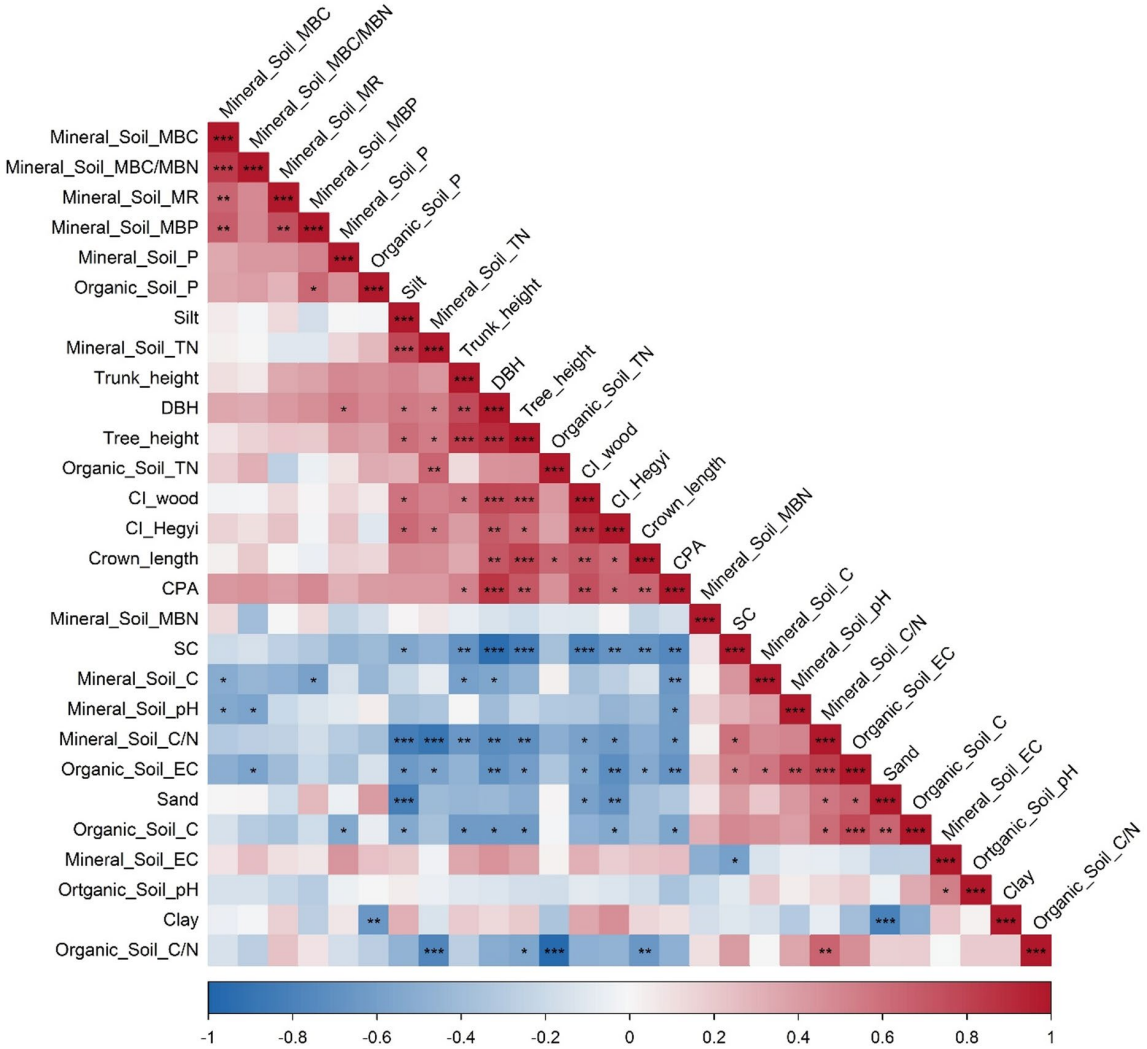
Pearson correlation heatmap illustrating relationships among canopy structural attributes, competition intensity, and soil properties in pure oriental beech stands. Colors indicate the strength and direction of correlations (red = positive, blue = negative), with color intensity proportional to the correlation coefficient (*r*). The diagonal represents self-correlations (*r* = 1). Asterisks denote statistically significant correlations (^*^ *p* < 0.05; ^**^ *p* < 0.01; ^***^ *p* < 0.001).

In pure oriental beech stands, strong and coherent correlation patterns were observed among canopy structural attributes, competition intensity, and soil properties (Fig. 4). Canopy size–related variables, including DBH, crown projection area (CPA), crown length, tree height, and trunk height, were positively and significantly correlated with each other (*r* = 0.55–0.92, *p* < 0.001). The wood-density–based competition index (CI_wood_) and the spatially explicit Hegyi competition index (CI_Hegyi_) showed strong positive correlations with DBH and CPA (*p* < 0.001), reflecting increased competitive interactions in structurally developed stands. Soil biological indicators, particularly microbial biomass carbon (MBC), microbial biomass nitrogen (MBN), and their ratios, exhibited positive associations with canopy structural variables and both competition indices (*p* < 0.01), whereas mineral soil C/N and organic layer C/N ratios were negatively correlated with canopy size metrics and competition intensity (*p* < 0.05). Mineral soil total nitrogen and phosphorus showed moderate positive correlations with canopy structure and microbial biomass pools. In contrast, soil texture components displayed opposing relationships, with sand content negatively correlated and clay content positively correlated with soil carbon pools, microbial indicators, and canopy structural variables (*p* < 0.05). EC and soil pH exhibited weaker and less consistent associations across variables.

**Fig. 5:**
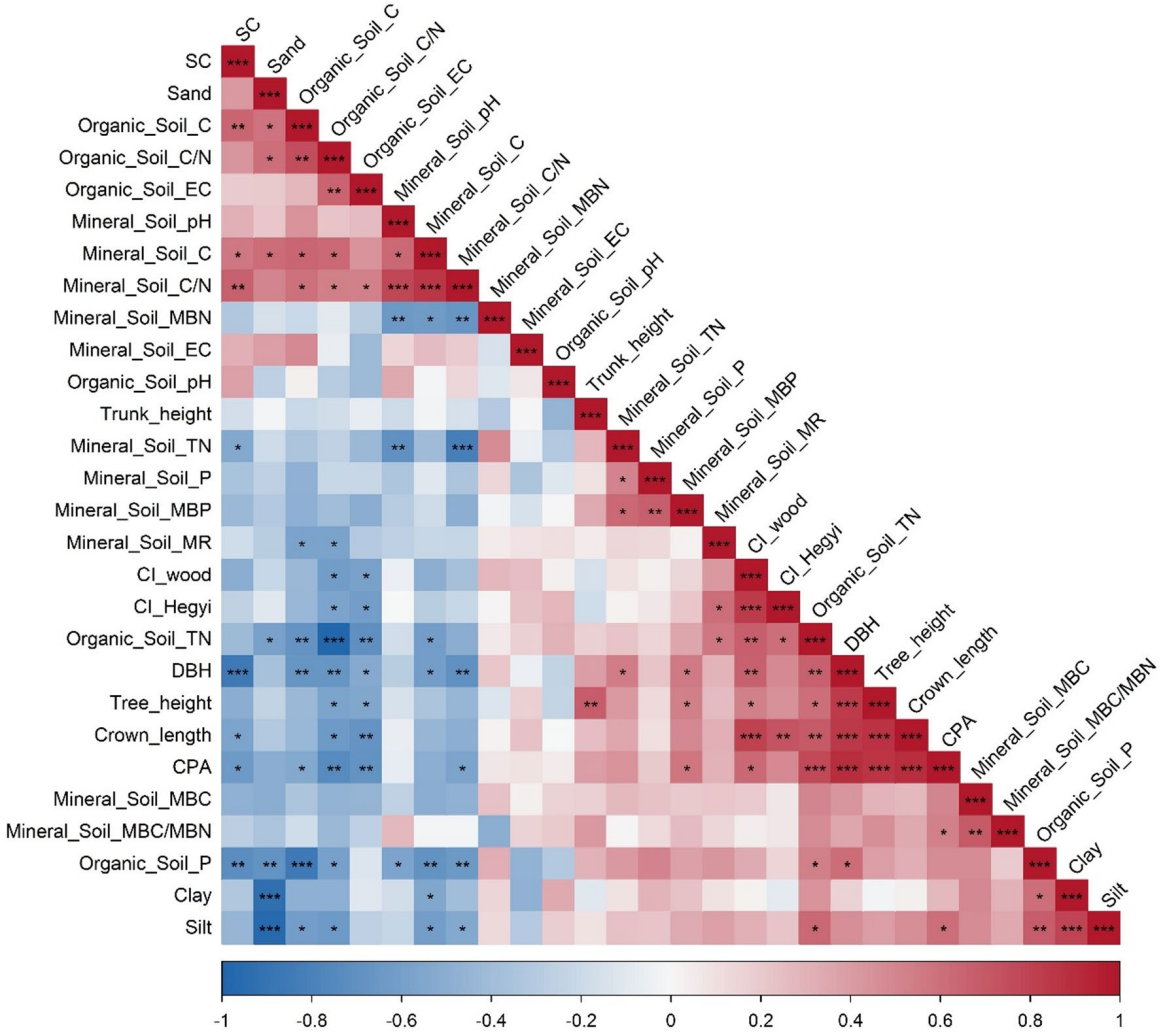
Pearson correlation heatmap showing relationships among canopy structural attributes, competition intensity, and soil properties in mixed oriental beech stands. Colors represent the direction and magnitude of correlations (red = positive; blue = negative), with darker shades indicating stronger relationships. The diagonal indicates self-correlations (*r* = 1). Statistically significant correlations are denoted by asterisks (* *p* < 0.05; ** *p* < 0.01; *** *p* < 0.001).

In mixed oriental beech stands, correlation patterns among canopy structure, competition intensity, and soil properties were more heterogeneous than those observed in pure stands (Fig. 5). Canopy structural variables, including DBH, crown projection area (CPA), crown length, tree height, and trunk height, remained positively correlated (*r* = 0.48–0.88, *p* < 0.001); however, the strength of these associations was generally weaker and more variable than in pure stands. The wood-density–based competition index (CI_wood_) and the spatially explicit Hegyi competition index (CI_Hegyi_) showed strong positive correlations with DBH and CPA (*p* < 0.001). Soil biological indicators, particularly microbial biomass carbon (MBC), microbial biomass nitrogen (MBN), and microbial biomass stoichiometric ratios, exhibited stronger and more widespread positive correlations with canopy structural variables and both competition indices than in pure stands (*p* < 0.01). In contrast to pure stands, mineral soil nutrient pools (total N and P) and organic layer nutrient concentrations showed more consistent positive associations with canopy size metrics and microbial activity. Soil texture effects were also more pronounced in mixed stands, with sand content showing strong negative correlations and clay and silt contents showing positive correlations with soil carbon pools, microbial biomass, and canopy structural attributes (*p* < 0.05). EC and soil pH displayed clearer associations with both microbial and canopy variables than in pure stands (Figs. 4 and 5). The strong positive correlation between CI_wood_ and CI_Hegyi_ further indicates consistency between stand-level and spatially explicit competition metrics (Figs. 4 and 5).

### Multivariate coupling of canopy structure, competition intensity, and soil properties (RDA)

**Fig. 6:**
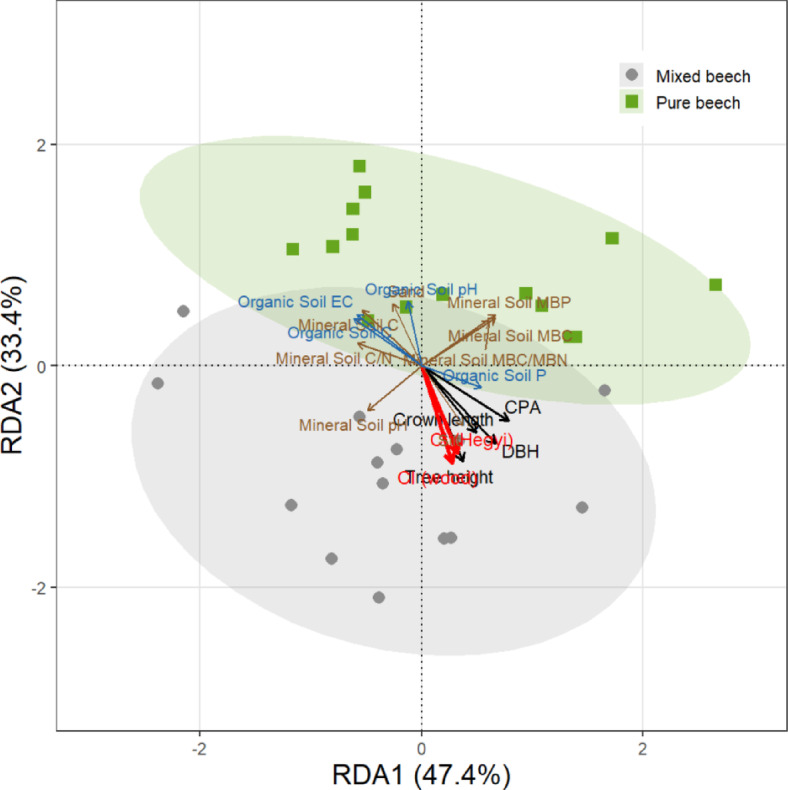
Redundancy analysis (RDA) biplot illustrating relationships between canopy structural attributes, competition intensity, and soil properties across pure and mixed virgin oriental beech stands. Structural variables (DBH, tree height, crown length, crown projection area [CPA]) are shown as black vectors, mineral soil variables as brown vectors, competition indices as red vectors, and organic layer variables as dark blue vectors. Points represent individual plots, with shaded ellipses indicating 95% confidence regions for mixed (gray) and pure (green) stands. The first two RDA axes explain 47.4% (RDA1) and 33.4% (RDA2) of the constrained variation, respectively. Vector length and direction indicate the strength and sign of relationships with the ordination axes.

To examine multivariate relationships between forest structure, competition intensity, and soil properties, redundancy analysis (RDA) was conducted using canopy structural attributes (DBH, tree height, crown length, crown projection area, and both the wood-density–based competition index (CI_wood_) and the spatially explicit Hegyi competition index (CI_Hegyi_)) as explanatory variables and a combined set of mineral and organic soil properties as response variables. The first two RDA axes explained 80.8% of the total constrained variance (RDA1 = 47.4%, RDA2 = 33.4%) (Fig. 6). The overall RDA model was statistically significant (*F* = 2.37, *p* = 0.001; 999 permutations). Forward permutation tests indicated that the first (RDA1: *p* = 0.001) and second axes (RDA2: *p* = 0.003) were significant, whereas subsequent axes were not. Along RDA1, pure beech stands were clearly separated from mixed stands. RDA1 axis represents a gradient of increasing canopy structural development and competition intensity, ranging from structurally complex, competition-dominated pure stands to more heterogeneous, lower-competition mixed stands. Pure stands were positioned toward higher values of tree height, DBH, crown length, crown projection area, as well as both competition indices, and were associated with higher mineral soil microbial biomass carbon and nitrogen, mineral soil total nitrogen, and organic layer nitrogen content. In contrast, mixed stands were located on the opposite side of RDA1 and were associated with higher mineral soil pH, sand content, and organic layer carbon and C/N ratios (Fig. 6). Mixed-stand plots also occupied a broader area of the ordination space relative to pure stands. RDA2 represented secondary variation primarily associated with soil texture and acidity, with mineral soil pH and sand content opposing organic and mineral nitrogen pools. RDA2 represented secondary variation primarily associated with soil physicochemical properties, particularly soil texture and acidity, reflecting variation in nutrient availability and edaphic conditions across plots. This axis indicates variation in nutrienty availability and edaphic conditions across plots. Both stand types overlapped along this axis, although pure stands generally occupied lower RDA2 scores. Notably, CI_wood_ and CI_Hegyi_ showed similar directional loadings in the ordination space (Fig. 6), indicating consistency between stand-level and spatially explicit representations of competition intensity.

## Discussion

This study demonstrates that stand composition reorganizes the coupling among canopy structure, competition intensity, and soil processes into distinct ecosystem functional configurations, as revealed by integrated structural, competition, and multivariate analyses. Although several observed patterns—such as higher competition intensity in pure stands and compositional effects on soil properties—are consistent with previous studies, the key advance here is the integration of these components within a single structural–functional framework. By jointly analyzing canopy structure, two complementary competition indices (CI_wood_ and CI_Hegyi_), and soil properties, we show that pure and mixed beech stands represent fundamentally different ecosystem states rather than points along a single structural gradient. This conclusion is supported by consistent patterns across univariate comparisons, correlation structures, and RDA.

Competition intensity and its structural organization differed fundamentally between pure and mixed stands, as indicated by consistent increases in both CI_wood_ and CI_Hegyi_ in pure stands. Pure beech stands supported larger trees with longer crowns and higher competition indices, indicating a dominance-driven system in which large individuals exert disproportionate control over resource capture, consistent with patterns reported for European beech forests^12,59^. The agreement between CI_wood_ and CI_Hegyi_ shows that competitive interactions are reinforced across both functional (trait-based) and spatial (neighbor-driven) dimensions. Although both stand types exhibited similarly high DBH variability, competition in pure stands was centralized around dominant trees, consistent with size-asymmetric competition and strong vertical canopy stratification, as reported in pure beech systems^12^,^59^. Because saplings and full spatial structure were not included, these results should be interpreted as structural differentiation rather than direct evidence of competitive asymmetry.

In contrast, mixed stands exhibited a redistribution of competition rather than a reduction in competition intensity, reflecting species-driven differences in canopy organization, as evidenced by lower competition indices and more evenly distributed tree sizes in mixed stands. Mixed stands had lower competition indices and smaller average tree size, yet maintained comparable size variability, indicating a more evenly distributed competitive structure. Competition was dispersed among individuals rather than centralized in dominant trees, consistent with evidence that species mixtures reduce size inequality and moderate canopy stratification^12^. This redistribution likely arises from species-specific differences in crown architecture and resource acquisition strategies, which reduce direct overlap among individuals and moderate competitive asymmetry. These patterns may also be interpreted in the context of conspecific negative density dependence (CNDD), where high conspecific density in pure stands may intensify intra-specific interactions, whereas species mixtures may reduce such density-dependent effects; however, this mechanism was not explicitly tested in this study and should be interpreted cautiously.

These differences in competition structure were directly reflected in soil processes, as shown by significantly higher microbial activity and nutrient concentrations in mixed stands (Fig. 3). These biologically active conditions indicate enhanced nutrient cycling in mixed stands, consistent with the observed increases in microbial biomass and respiration (Fig. 3). Such biologically active mineral soils are widely recognized as key targets in forest restoration aimed at rebuilding nutrient cycling and soil functioning following disturbance. This pattern directly agrees with evidence showing that mixtures of beech with nutrient-richer broad-leaved species (e.g., common hornbeam and velvet maple) promote higher biological activity and soil fertility through faster litter decomposition and improved forest-floor quality^60–62^. Higher litter quality and compositional heterogeneity in mixed stands likely enhance microbial stimulation and accelerate nutrient cycling, thereby increasing mineral soil N, P, and microbial activity^63,64^. Conversely, pure beech exhibited a more conservative soil functioning pattern, characterized by lower microbial activity and reduced nutrient availability despite greater canopy dominance. Consistent with these patterns, pure stands exhibited lower microbial activity and biomass, suggesting a decoupling between aboveground dominance and belowground biological activity. This pattern is consistent with the higher recalcitrance of beech litter, which typically exhibits lower nutrient contents and higher C/N ratios, limiting microbial activity and slowing nutrient turnover^60,62,64^. These results indicate that canopy dominance does not necessarily enhance soil functioning, but may instead promote carbon retention and reduced nutrient cycling rates, representing an alternative ecosystem strategy shaped by stand composition.

Organic-layer responses were more selective and reflected short-term litter-driven processes rather than integrated soil functioning. Mixed stands exhibited higher organic-layer pH and nitrogen concentrations, whereas pure beech stands were characterized by higher C/N ratios (Fig. 2), indicating faster litter decay and more efficient nitrogen turnover in mixed stands, driven by higher forest-floor quality and greater nutrient availability^61,63^. In contrast, higher C/N ratios in pure beech stands imply stronger nutrient limitation of soil microorganisms, where decomposition is constrained more by N availability than by carbon supply^65^. The absence of significant differences in organic carbon and phosphorus concentrations suggests that total organic matter inputs were broadly comparable across stand types. These results indicate that early-stage decomposition processes in this system are primarily controlled by litter quality and composition rather than total organic matter inputs.

Stand composition altered not only the magnitude of relationships among variables, but also the organizational structure of canopy–soil coupling, as demonstrated by correlation analyses. In pure stands, Pearson correlations revealed a coherent and tightly coupled network in which canopy structural attributes and competition intensity were strongly associated with mineral soil nutrients and microbial biomass (Fig. 4). Larger tree size, greater crown development, and higher wood-density–based competition intensity consistently coincided with higher microbial biomass C and N and lower soil C/N ratios, indicating a system governed by a dominant structural gradient linking canopy organization to soil processes. Similar structurally controlled canopy–soil feedbacks have been reported in primary European beech forests^66^. In contrast, mixed stands exhibited weaker and more heterogeneous correlation structures (Fig. 5), indicating a more distributed control of soil processes. Although canopy structural variables remained intercorrelated, their relationships with soil properties were less centralized and more variable (Fig. 5). Microbial indicators showed broader associations with both canopy and soil variables, indicating that belowground processes are influenced by multiple interacting drivers rather than being dominated by canopy structure alone, consistent with findings in oriental beech forests^67,68^. This indicates that stand composition modifies not only ecosystem properties, but also the pathways through which canopy structure and competition influence soil processes.

RDA further confirmed that pure and mixed stands represent distinct ecosystem functional states in multivariate space (Fig. 6). Pure stands aligned with larger canopy dimensions, higher competition intensity, and elevated microbial biomass C and N, whereas mixed stands were associated with higher soil pH and organic-layer C and C/N ratios, consistent with patterns observed in mixed beech systems^69,70^. The clear separation along RDA1 therefore reflects a transition between structurally controlled and composition-driven ecosystem organization rather than a simple structural gradient. The similar directional loadings of CI_wood_ and CI_Hegyi_ further indicate strong consistency between trait-based and spatially explicit representations of competition intensity. Although the observed patterns indicate strong linkages among canopy structure, competition intensity, and soil properties, these relationships are based on correlation and ordination analyses and therefore do not establish causality. Future studies could build on this framework using structural equation modeling (SEM) to explicitly test hypothesized causal pathways and quantify direct and indirect effects.

### Management implications

Results from this study show that pure and mixed oriental beech stands represent fundamentally different functional systems, even when they occur under identical climatic, topographic, and developmental conditions. This distinction is directly supported by higher competition intensity (CI_wood_ and CI_Hegyi_) and larger canopy dimensions in pure stands, versus higher microbial biomass and respiration in mixed stands (Fig. 3). For forest restoration and close-to-nature management, this means that stand composition defines alternative structural–functional reference conditions, rather than minor variations of a single system. Pure stands are characterized by strong vertical canopy stratification and dominance-driven competition, whereas mixed stands maintain lower competition intensity but support more biologically active soils.

For forest managers, these differences imply that stand composition decisions directly influence both aboveground structure and belowground functioning. In pure stands, the strong coupling between canopy structure, competition intensity, and soil properties suggests that structural attributes (e.g., tree size and crown development) can be used as indicators of system functioning. In contrast, mixed stands require consideration of both compositional diversity and soil biological indicators, as soil processes are not solely controlled by canopy structure. Because these patterns were identified in virgin forests, they provide empirical reference conditions for evaluating management outcomes. Management strategies that simplify species composition are likely to increase competition intensity and shift systems toward structurally dominated conditions, whereas maintaining species mixtures may enhance microbial activity and nutrient cycling. Incorporating indicators such as competition indices (CI_wood_ and CI_Hegyi_) and microbial biomass into monitoring frameworks can therefore improve the alignment between structural targets and soil recovery.

More broadly, these findings suggest that management aimed at maintaining species mixtures and structural heterogeneity may better support active nutrient cycling and soil biological functioning, although the strength of these responses may vary with species composition and site conditions. Importantly, the results indicate that trade-offs may exist between structurally dominated systems (pure stands) and biologically active systems (mixed stands), and management objectives should explicitly consider which functional state is desired.

## Conclusion

This study demonstrates that stand composition strongly influences the coupling between canopy structure, competition intensity, and soil processes in virgin oriental beech forests. Under identical climatic, topographic, and developmental conditions, pure beech stands formed dominance-driven canopies with strong vertical canopy stratification and high competition intensity (as consistently indicated by higher CI_wood_ and CI_Hegyi_ values), whereas mixed stands consistently supported more biologically active mineral soils characterized by higher microbial biomass and respiration. These results demonstrate that pure and mixed stands represent distinct structural–functional states, rather than variations along a single gradient, under the conditions examined in this study.

Multivariate analyses further revealed that the organization of canopy–soil linkages differs systematically between stand types, with pure stands exhibiting centralized, structurally regulated coupling and mixed stands showing more distributed and composition-driven patterns. The clear separation of stand types in RDA and the consistency between competition indices indicate that stand composition modifies not only ecosystem properties, but also the pathways linking canopy structure, competition intensity, and soil processes. Although this study is based on a rare virgin forest system, the observed structure–function relationships provide an empirical reference for understanding how stand composition reorganizes ecosystem functioning, while acknowledging that their magnitude and direction may vary across regions, species compositions, and management histories. These findings highlight stand composition as a key lever for managing structural-functional trade-offs in temperate virgin beech forests, with species mixtures enhancing biological functioning and pure stands promoting structurally regulated, competition-driven dynamics. 

## Supplementary Information

Below is the link to the electronic supplementary material.

Supplementary Material 1

## Data Availability

The datasets generated and analyzed in this study are available from the corresponding authors upon request.
