# Supplementary material for: Stand composition shapes canopy structure, competition, and soil properties in virgin oriental beech forests
Source: Sci Rep. 2026 Apr 14;16:17299. doi: 10.1038/s41598-026-48111-3 (PMC13234411; doi:10.1038/s41598-026-48111-3)
Supplement: Supplementary file 1 — Supplementary Material 1 [file 41598_2026_48111_MOESM1_ESM.docx]

**Fig. S1.** Diameter class distribution (5-cm intervals) of trees in pure and mixed oriental beech forests based on stem density (stem ha^-1^).
